# Emotion inference in conversations based on commonsense enhancement and graph structures

**DOI:** 10.1371/journal.pone.0315039

**Published:** 2024-12-11

**Authors:** Yuanmin Zhang, Kexin Xu, Chunzhi Xie, Zhisheng Gao

**Affiliations:** 1 China Unicom (Sichuan) Industrial Internet Co. Ltd., Chengdu, Sichuan, People’s Republic of China; 2 School of Computer and Software Engineering, Xihua University, Chengdu, Sichuan, People’s Republic of China; Tongji University, CHINA

## Abstract

In the task of emotion inference, a common issue is the lack of common sense knowledge, particularly in the context of dialogue, where traditional research has failed to effectively extract structural features, resulting in lower accuracy in emotion inference. To address this, this paper proposes a dialogue emotion inference model based on Common Sense Enhancement and Graph Model (CEICG). This model integrates external common sense with graph model techniques by dynamically constructing nodes and defining diverse edge relations to simulate the evolution of dialogue, thereby effectively capturing the structural and semantic features of the conversation. The model employs two methods to incorporate external common sense into the graph model, overcoming the limitations of previous models in understanding complex dialogue structures and the absence of external knowledge. This strategy of integrating external common sense significantly enhances the model’s emotion inference capabilities, improving the understanding of emotions in dialogue. Experimental results demonstrate that the CEICG model outperforms six existing baseline models in emotion inference tasks across three datasets.

## 1 Introduction

Emotion is an individual’s attitude and experience toward objective events during psychological processes. Humans are easily influenced by emotions, leading to a series of subtle decisions that shape personal habits, career planning, and social relationships. Emotion analysis can utilize various features, such as text features, facial features [1], and behavioral features [2], to understand people’s emotional characteristics, which hold significant importance across various fields [3]. Providing robots with emotional skills has been a key focus in this area, with one important aspect being the ability of robots to actively recognize emotions. On the other hand, emotion analysis enables robots to adapt to users’ emotional states and provide more appropriate emotional responses, enhancing user experience in fields such as medical services [4], counseling services [5], and educational services [6]. Moreover, with societal development, human communication has shifted from traditional audiovisual methods to text-based methods, such as online customer support systems. This shift presents challenges in obtaining emotional analysis data that was previously reliant on audiovisual cues. Therefore, analyzing emotions in dialogue text in the absence of audiovisual data contributes to expanding the research domain of emotion analysis.

Traditional emotion analysis methods are primarily based on statistical models and machine learning techniques [7–10]. However, these methods often only support binary classification, exhibit poor performance, and have weak generalization capabilities, as they mainly rely on objective patterns in the data to predict emotions. This reliance results in the need for extensive computation to build high-accuracy models using traditional emotion analysis methods, making it even more challenging for large-scale datasets. Combining deep learning with dialogue emotion analysis is a promising approach, as it enhances the model’s generalization ability while simulating human behavior to capture emotional features in vast amounts of data. It can also learn features that are often overlooked but highly relevant to emotion analysis results [11]. This approach aims to enhance machines’ understanding of human emotions and provide robust monitoring of various negative emotions in society.

Emotion prediction is a popular topic. Compared to traditional methods that only identify the emotions of specific sentences, the main goal of emotion prediction is to forecast the user’s emotional state at the next moment. Due to the dependency on emotional statements and the diversity of emotions, recognizing emotions during a conversation becomes increasingly challenging, as emotional changes correspond to variations in dialogue expressions. To accurately differentiate among several emotional categories within discourse, it is essential to consider the contextual features of past information. While this may be simple for humans, it poses a significant challenge for machines. Humans can grasp the implied meanings in language and context, such as sarcasm, humor, or puns. Different cultures have varying norms and customs for expressing and understanding emotions, and humans are able to accurately interpret emotions based on this background knowledge, which largely relies on sociocultural experience and contextual knowledge. Furthermore, previous research models typically handle individual sentences rather than entire conversations [12, 13]. Consequently, they are limited in their ability to understand linguistic relevance across multiple dialogue turns and may fail to capture subtle linguistic cues and contextual changes in ongoing conversations [14, 15]. To address this, we introduce external common knowledge through an external knowledge base as auxiliary information to enhance the representational performance of discourse. Additionally, to better capture the relationships between dialogues, we design a graph model to represent conversations, aiming to improve the understanding of dialogue features and enrich the variables extracted. The primary research contributions of this paper are as follows:

(1) The CEICG model based on external knowledge was proposed. This model draws on the principles of deep learning, with sentences in the dialogue system being initially encoded and emotional features fine-tuned using RoBERTa. Feature extraction was subsequently performed using LSTM. A graph model was then constructed based on sentence nodes to capture the structural features within the dialogue. Furthermore, two methods were employed to extract knowledge from the sentences, which was integrated as auxiliary information into the graph model. This approach of incorporating external common knowledge significantly enhanced the model’s emotional reasoning capabilities. Finally, Graph Convolutional Networks (GCN) were utilized for node updates and feature extraction within the graph model, providing a basis for emotional reasoning.
(2) Experiments were conducted across three distinct datasets, with the results being compared against existing baseline methods. The experimental findings indicate that, in comparison to traditional methods, the proposed model demonstrates significant advantages, validating its advanced and practical efficacy in addressing dialogue emotion reasoning tasks.

## 2 Related work

Emotions are regarded as patterns related to the environment, encompassing a series of interconnected events, including environmental stimuli, psychological changes, self-perception, and behavioral impulses. It has been demonstrated that emotions serve as a means to improve human social relationships and enhance our adaptability to the ever-changing environment [16]. Researchers have categorized emotions based on various emotional theories. Among these, the most well-known classification was proposed by Ekman [17], who identified six basic emotions: surprise, sadness, fear, joy, disgust, and anger, which are viewed as core patterns of emotional responses. Subsequent studies have expanded this classification to include eight categories [18], fifteen categories [19], and so on.

Emotion prediction has long been a popular topic. Hasegawa et al. [10] predicted the emotions of recipients in online dialogues using statistical methods within the context of two-turn dialogues. Zhang et al. [20] introduced a novel method for interactive emotional learning, designing a Transformer-based variational learning network to learn the response distribution between dialogues to predict future emotions. Soujanya et al. [21] explored spatial and temporal attention, as well as the parallel/sequential arrangement of spatial and temporal attention modules, to enhance emotion prediction performance by integrating information about emotions and emotional changes. Li et al. [22] proposed an interactive emotional model that summarizes and utilizes the characteristics of dialogue texts to effectively extract the historical emotional features and emotional change features of dialogue users in an interactive manner, predicting the speaker’s future emotions based on previous utterances. Radhika et al. [23] noted that emotional labels themselves might convey information, as these labels can guide the model’s attention distribution upon input. The emotions triggered by an event are often interrelated and exhibit a certain homogeneity. Sun et al. [24] combined reinforcement learning and emotional editing mechanisms for emotion prediction during response tasks, resulting in replies that are both logically and emotionally relevant. Liu et al. [25] proposed a simple and effective dialogue emotion prediction model based on relationship extraction, utilizing self and other dialogues to extract and incorporate relevant emotional dependencies, predicting their emotions without the emotional context of the current utterance, and measuring the similarity between the emotional distribution and the emotion prediction distribution using KL divergence. Enas et al. [26] modeled the three inherent dimensional relationships that evoke emotions in dialogues, merging them into two deep neural network architectures: one being a Graph Convolutional Network model and the other a sequence network model, aimed at capturing the network formation and discourse sequence features within dialogues. Gopendra et al. [27] proposed an end-to-end model that directly models category labels using label embedding techniques. This model can handle text, audio, and visual features concurrently, integrating various modalities through a cross-attention network to identify emotions and intentions in multimodal environments.

The objective of emotion reasoning tasks is primarily to infer how events evoke the emotions of characters in stories. In dialogue systems, researchers have redefined this task. Li et al. [28] redefined emotional reasoning within the dialogue process, where the main goal is to predict how utterances influence the listener’s emotions without knowing any responses from the listener, specifically predicting their emotional state in the next turn. However, the challenge lies in the unknown future dialogue content, which limits the ability to directly infer future emotional states. Particularly, understanding how emotions propagate among participants in dialogues is key to achieving accurate emotional reasoning. By accurately understanding and predicting the emotional changes of dialogue listeners, dialogue systems can communicate more naturally and effectively with users, thereby playing a crucial role in providing customer support, mental health counseling, and personalized recommendations. This enhancement in emotional perception capabilities lays the foundation for constructing a more human-like human-computer interaction experience. In existing research on emotional reasoning, with the development of deep learning, Li et al. [28] modeled the propagation of emotional states between participants by deeply analyzing dialogue history. A deep learning model based on address-aware modules and ensemble strategies was proposed, which automatically identifies whether participants maintain their previous emotional states or are influenced by other participants in the dialogue, thus exhibiting corresponding emotional responses in the next round of dialogue. Wang et al. [29] introduced an innovative global and local modeling technique that combines the ability of RNNs to process sequential data with the powerful capabilities of pre-trained language models (PLMs) for deep language understanding. Through this combination, the model captures dialogue dynamics while extracting and utilizing knowledge from extensive contexts. Specifically, the entire dialogue history is used as input to the PLM, employing a contextual learning mechanism to generate knowledge regarding the dialogue context, thereby providing a new approach for emotional reasoning. Narayana et al. [30] inferred short-term emotional states by focusing on long-term emotional influences and their changes, rather than relying on traditional contextual cues such as background scenes, locations, or social actors. This approach shifts away from the previous unidimensional methodology by integrating video information for multimodal emotional reasoning.

External knowledge bases refer to collections of knowledge relied upon by a system or program that exist outside the system itself, encompassing relevant definitions, concepts, examples, rules, experiences, and other information. In the fields of artificial intelligence and natural language processing, external knowledge bases are frequently used to supplement model training data, thereby enhancing the model’s understanding and reasoning capabilities. Numerous common-sense knowledge bases exist in current research, including Event2Mind [31] and ATOMIC [32], which contain over 877k instances of everyday common-sense knowledge organized into variable-type if-then relationships with strict logical connections. ConceptNet [33] is a semantic network that includes concept-level relationship common sense, aiding machines in understanding the meanings of words. SenticNet [34] is an emotional lexicon widely used in sentiment analysis tasks. COMET [35] is a generative model trained on ConceptNet and ATOMIC, which generates richer and more diverse common-sense knowledge that is not present in the original knowledge bases based on original knowledge. To address the issue of machines being unable to rely on context and common-sense knowledge like humans, Zhou et al. [13] employed a method of retrieving common-sense knowledge relevant to user sentences and structuring each knowledge graph as a separate entity for encoding. This process strengthens the semantic meaning of sentences through the use of a static graph attention mechanism, thereby obtaining responses rich in information. Zhong et al. [36] developed a knowledge-enhanced transformer model that adopts a hierarchical self-attention mechanism to analyze contextual utterances and dynamically introduces external common sense through context-aware emotional graph attention mechanisms, significantly improving the accuracy of emotion recognition. Li et al. [37] proposed a knowledge integration strategy for integrating common-sense knowledge related to dialogues generated by event-based knowledge graphs. Ghosal et al. [38] and his team developed a novel framework named COSMIC that integrates various common-sense features, including mental states, event themes, and their causal relationships, using this information to study the interactions between participants in dialogues.

Currently, most research in dialogue systems focuses on the classification of emotional polarity as positive, negative or neutral at the utterance level for both dialogue participants [10, 22], with a lack of consideration for emotional reasoning work related to external common sense.

## 3 Methodology

### 3.1 Definition of the problem

Let *U* = {*μ*_1_, *μ*_2_, …, *μ*_*T*_} be a set of dialogues, where *T* is the length of the dialogue, such as *μ*_*t*_ represents the utterance spoken by the speaker at time *t*, with each utterance consisting of several words. The task of emotion inference is to deduce the emotional reaction of the recipient of the last utterance *μ*_*t*_ in the dialogue set, without knowing any information after *μ*_*T*_, by utilizing the entire dialogue’s utterance information. Specifically, it is defined as *E*_*T*_ ∼ *P*(*E*_*T*_|(*μ*_1_, *μ*_2_, …, *μ*_*t*_, …, *μ*_*T*_)), where *E*_*T*_ is the predicted emotional probability distribution corresponding to the recipient.

**Fig 1:**
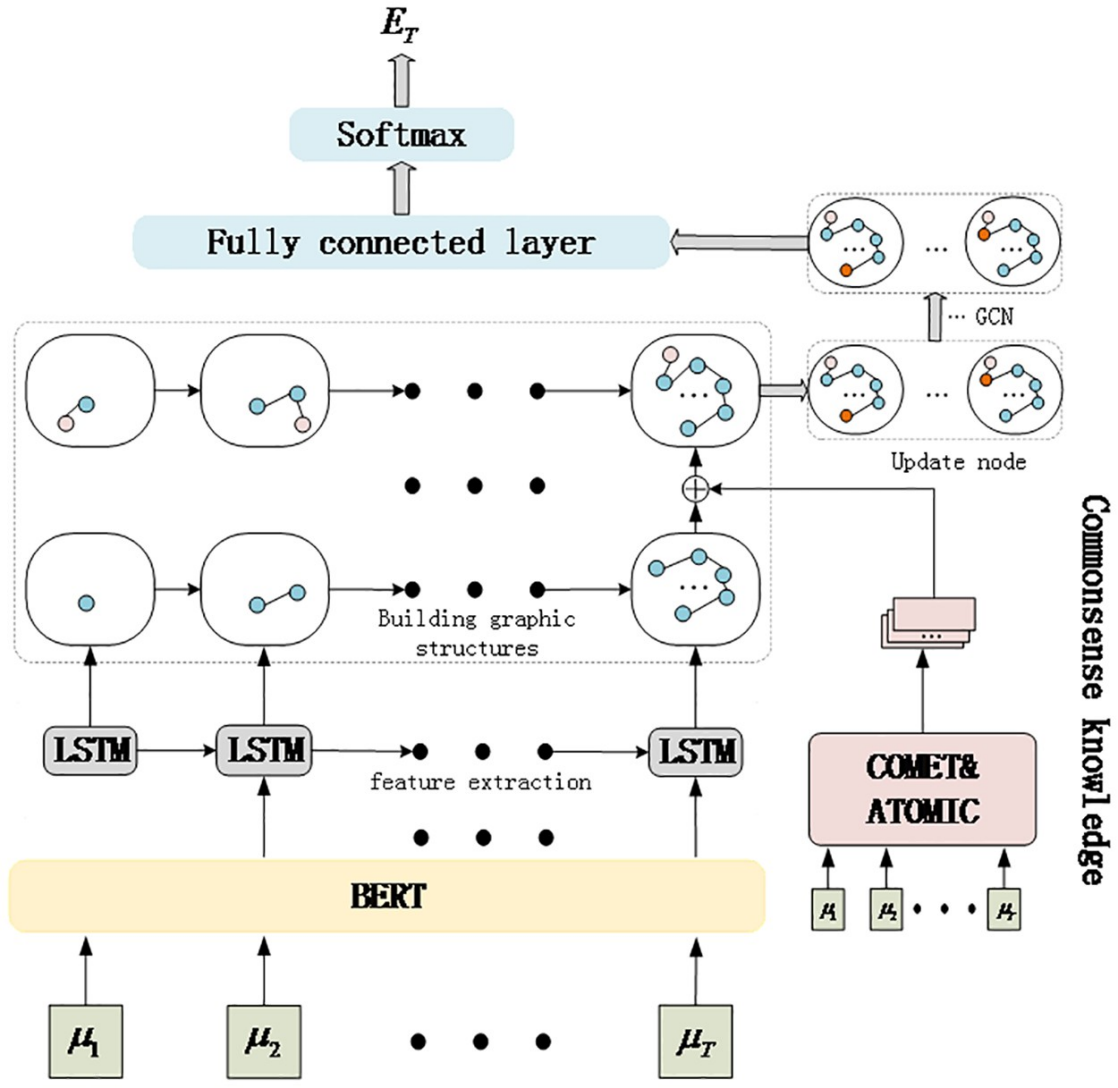
The architecture of CEICG model.

This paper proposes a dialogue emotion inference model based on commonsense enhancement and graph modeling (CEICG). Based on the idea that each utterance in a dialogue should have a certain connection with previous utterances, we not only consider the relevant sequential information but also the rationality of information change over time. To achieve this, we transform the dialogue process into a graph structure for storage, and then use GCN [39] to update the node information within the graph. This node information can be viewed as emotional information derived from external commonsense and dialogue sequence features. Additionally, we integrate external commonsense as auxiliary information into the model, and finally obtain the corresponding emotional probability distribution through a classifier. The structure of the proposed CEICG model is shown in Fig 1. The proposed CEICG model comprises three main components: emotion feature extraction, construction of the dialogue graph with integrated external commonsense, and emotion inference.

### 3.2 Emotion feature extraction

The process primarily includes two main steps: word embedding and feature extraction.

**(1) Word Embedding**

Word embedding is a natural language processing technique that maps words or phrases from a vocabulary into a high-dimensional space composed of numerical vectors. Traditional word representation methods, such as One-hot Encoding, generate vectors with lower dimensions. However, most elements in these vectors are zeros, making them inefficient and unable to effectively capture relationships between words. RoBERTa (Robustly optimized BERT approach) is an improved version of BERT, introduced by Facebook AI in 2019. RoBERTa enhances BERT by training on larger datasets for longer periods and removing the Next Sentence Prediction (NSP) task during the pre-training phase. This allows RoBERTa to capture richer linguistic features and subtle semantic differences. Therefore, we choose RoBERTa as the encoder to generate dynamic word embeddings, meaning that the same word will have different embeddings in different contexts. This characteristic enables our embeddings to handle polysemy and context-related semantics. We obtain the vector representation of each sentence from the last layer of the encoder. The encoding process is represented as follows:

$$\begin{array}{c} v_{1},v_{2},\ldots ,v_{T}=RoBERTa\left(\mu_{1},\mu_{2},\ldots ,\mu_{T}\right). \end{array}$$ (1)

Here, *v*_1_, *v*_2_, …, *v*_*T*_ are the representations of the corresponding utterances *μ*_1_, *μ*_2_, …, *μ*_*T*_.

**(2) Feature extraction**

In real conversations or multi-turn dialogue environments, the sentiment of the text may change as the dialogue progresses. In other words, the sentiment at time *t* can change due to the accumulation of sentiment from previous conversations. Therefore, the introduction of LSTM will help the model better track and understand these dynamic changes in sentiment. For example, considering that the sentiment tendencies of the text may be influenced by distant phrases, LSTM effectively retains this important information through its gating mechanisms, even if there is a long interval between them. At the same time, the forget gate of LSTM allows the model to “forget” unimportant or irrelevant information, which helps the model focus on information more critical to the current task. For a specific utterance representation *v*_*t*_, we use it as the input to LSTM to obtain the sentiment features of each sentence. This process is formalized as follows:

$$\begin{array}{c} \left(h_{t},c_{t}\right)=LSTM\left(v_{t},\left(h_{t-1},c_{t-1}\right)\right) \end{array}$$ (2)

Here, *h*_*t*_ denotes the hidden state at time *t*, *c*_*t*_ represents the cell state at time *t*, *v*_*t*_ is the input at time *t*, *h*_*t*−1_ is the hidden state at time *t* − 1, and *c*_*t*−1_ is the cell state at time *t* − 1. In our experiments, the initial hidden state is set to 0.

### 3.3 Constructing dialogue graphs integrating external knowledge

#### 3.3.1 Constructing dialogue graph

Given a set of conversations *U* = {*μ*_1_, *μ*_2_, …, *μ*_*t*_, …*μ*_*T*_}, which are transformed into conversation representation features {*h*_1_, *h*_2_, …, *h*_*t*_, …*h*_*T*_} through an emotion feature extraction component. Let *H*_*t*_ = {*h*_1_, *h*_2_, …, *h*_*t*_} be the set of nodes in the graph at time *t*, and let *γ*_*t*_ ← {{*h*_*i*_, *h*_*j*_}|*h*_*i*_, *h*_*j*_ ∈ *H*_*t*_, *h*_*i*_ ≠ *h*_*j*_} be the set of relational edges. The graph *G*_*t*_(*H*_*t*_, *γ*_*t*_) can then be used to represent the dialogue state at time *t*, where (*h*_*i*_, *h*_*j*_) represents a relational edge between the nodes *h*_*i*_ and *h*_*j*_. To better represent the sequential information and relevant semantic information of the utterances, two different types of relational edges are designed:

(1) **Sequential Edge**: This edge type represents a clear sequence between two utterances. For example, if a speaker says utterance *h*_*i*_, and another speaker replies with utterance *h*_*j*_, there is a clear sequential relationship between them. We define this relational edge as a sequential edge and set its weight to 1.
(2)**Semantic Edge**: This type of relationship represents the case where there is no clear sequential relationship between two utterances. In this case, we calculate the edge weight between them using cosine distance. We then prune the edges with low weights to remove unnecessary information. In the next time step, we insert the node representing the new utterance into the current graph to construct a new graph, thereby reflecting the temporal sequence and semantic connections of the utterances throughout the dialogue.

#### 3.3.2 Updating dialogue graphs with external commonsense

External commonsense refers to knowledge that has been widely recognized and validated within a specific domain, including but not limited to linguistic rules, common sense judgments, cultural background, and behavior patterns in specific scenarios. By incorporating this knowledge into models, it can help them better understand the implicit emotions and intentions in dialogue content. For example, in a conversation with the sentence, “There will be heavy rain tomorrow, our game might be canceled,” analyzing the text based solely on surface emotions might make it difficult to accurately determine the speaker’s mood. However, if the model possesses commonsense knowledge related to games, such as understanding that people usually feel disappointed when a game is canceled, the model can more accurately infer that the speaker might be expressing regret or disappointment.

To achieve this, we extract commonsense knowledge for each utterance during the dialogue process and integrate it into a graph model. In previous studies, the social commonsense graph ATOMIC and the classification/lexical knowledge graph ConceptNet have proven to enhance machine understanding in emotion analysis within natural language research. Among them, ATOMIC provides a large number of “if-then” relationships covering various scenarios in daily life. This rich knowledge base can help NLP models understand the logic, causes, and effects behind human behavior, thereby enhancing the model’s contextual understanding ability. ATOMIC, by segmenting different types of social interactions and outcomes of human behavior, offers fine-grained social interaction commonsense, which aids NLP models in more accurately predicting the consequences of human actions, understanding the dynamics of interpersonal relationships, and predicting emotions and psychological states. ATOMIC contains a large number of commonsense knowledge tuples, consisting of a head phrase, relation, and attribute, such as: (“Someone is watching a movie alone at home,” xReact, “afraid”). The relationship types and their meanings in the ATOMIC knowledge base are shown in Table 1. Utilizing ATOMIC’s knowledge can improve a model’s generalization ability to unseen text, enabling the model to not only understand the text based on its literal meaning but also make inferences using underlying commonsense, thereby exhibiting better performance when handling new scenarios and tasks.

**Table 1:** Types of relations in the ATOMIC knowledge base.

Relationship Type	Relationship Description
xIntent	Represents the intention or purpose behind an individual (X) performing a certain action. This relationship answers the question, “Why did X do this?”
xNeed	Refers to the conditions or actions that an individual (X) needs to complete before the behavior occurs. This describes the prerequisites for the behavior to happen.
xAttr	Describes the attributes or characteristics that an individual (X) may exhibit after performing a certain action. This relationship reveals the impact of the behavior on the individual’s traits or state.
xEffect	Focuses on the consequences that an individual (X) directly experiences as a result of performing a certain action. This relationship describes the direct impact of the behavior.
xReact	Describes the internal feelings or reactions of an individual (X) after performing a certain behavior. This relates to changes in the individual’s emotional or psychological state.
xWant	Indicates what the individual (X) wants to do after the behavior, reflecting the desires or motivations following the behavior.
oEffect	Describes the impact on others or things as a result of the behavior of an individual (X). This relationship considers the consequences of the behavior from the perspective of others or the environment.
oReact	Represents the reactions or emotions of others or observers to the behavior of an individual (X). This relationship depicts the emotional responses of others to the behavior.
oWant	Describes the actions that others may want to take after observing the behavior of an individual (X). This reflects the motivations or desires of others as a response to the behavior.

With the development of deep learning technology, more and more research is focusing on how to utilize deep learning models, especially pre-trained language models like BERT and GPT, to automatically generate and enhance knowledge graphs. Compared to traditional methods of knowledge graph construction, which primarily rely on manual editing, generative knowledge graphs can expand and update knowledge bases more quickly, improving the efficiency and coverage of knowledge acquisition. Moreover, generative methods can significantly reduce the labor costs associated with building and maintaining knowledge graphs. For example, COMET, a deep learning model based on GPT and pre-trained on ATOMIC, automatically generates commonsense knowledge tuples from text, thereby constructing and expanding knowledge graphs. In addition to acquiring traditional knowledge, this approach also allows us to obtain less frequently considered but still useful knowledge for our tasks.

Therefore, we divide knowledge acquisition into two parts: knowledge querying and knowledge generation. First, for knowledge querying, we utilize ATOMIC to perform knowledge queries. We use the SBERT model, based on cosine similarity and BERT, to calculate the similarity between each utterance and the Head phrases in ATOMIC, obtaining the relationship attributes that are most similar to the Head phrases as the commonsense knowledge we query. In our research, we mainly select two relationships that are highly relevant to sentiment analysis: xReact and oReact, which represent how the subject feels after the event and how others feel after the event, respectively. These two attributes are crucial for extracting emotions. Next, in the knowledge generation part, we use COMET-ATOMIC to generate new commonsense knowledge. We input the utterance *μ*_*i*_ and the relationship types to be selected (xReact and oReact) into the model, and the model outputs the corresponding results based on the relationship types we selected. We use BiLSTM to extract the feature vectors of each relationship, obtaining the knowledge representation based on knowledge querying, *o*^A- xReact^ and *o*^A- oReact^, and the knowledge representation based on knowledge generation, *o*^C- xReact^ and *o*^C- oReact^. Finally, all the knowledge is fused together, as shown below:

$$\begin{array}{c} K_{i}=ReLU\left(W^{T}\left(o_{i}^{\text{A-xReact}}+o_{i}^{\text{A-oReact}}+o_{i}^{\text{C-xReact}}+o_{i}^{\text{C-oReact}}\right)+b\right), \end{array}$$ (3)

where $o_{i}^{\text{A-oReact}}$ represents the commonsense knowledge extracted from the utterance *μ*_*i*_, *W*^*T*^ is the learnable parameter matrix, and *K*_*i*_ is the feature obtained after fusing the commonsense knowledge related to the utterance *μ*_*i*_. Here’s an example to illustrate how to derive the xReact and oReact relational properties from a sentence and generate common sense knowledge, followed by the fusion process. For convenience, the process is named DGIEKGen:

**DGIEKGen Algorithm Example**

Assume the input sentence *i* is: “Vincent passed the exam”.

- Step 1: Encode the Sentence—Use SBERT to encode sentence *i*, obtaining a vector representation.
- Step 2: Encode Head Phrases—Select a series of Head phrases from the ATOMIC database, such as “someone achieves success” or “someone reaches a goal,” and encode them using SBERT.
- Step 3: Calculate Similarity—Compute the cosine similarity between the encoded sentence vector and each Head phrase vector.
- Step 4: Select the most similar Head phrase—Choose the Head phrase most similar to the sentence based on the calculated similarity values. For sentence *i*, the most similar Head phrase is “someone achieves success.”
- Step 5: Retrieve Common Sense Knowledge—Use the selected Head phrase to find corresponding common sense knowledge from the ATOMIC database. For example, the common sense for the Head phrase “someone achieves success” might be “achieving a goal through effort.”
- Step 6: Extract xReact and oReact—Extract xReact and oReact relational properties related to emotion analysis from the retrieved common sense. For instance, for the common sense “achieving a goal through effort.”,
  - *xReact*_*i*_ (Vincent’s reaction): “Vincent feels happy.”
  - *oReact*_*i*_ (others’ reaction): “Others feel proud.”
- Step 7: Generate New Common Sense Using COMET-ATOMIC. For example,
  - For *xReact*_*i*_, the new common sense generated by the COMET-ATOMIC algorithm is:
    *XGen*_*i*_: “Vincent feels proud because his efforts paid off.”
  - For *oReact*_*i*_, the common sense generated by the COMET-ATOMIC algorithm is:
    *OGen*_*i*_: “Others celebrate Vincent because he achieved a significant accomplishment.”
- Step 8: Use BiLSTM to extract the corresponding query-based and generation-based knowledge representations, and then perform fusion (Eq 3) to generate the features of *K*_*i*_. Here,
  -
$o_{i}^{\text{A-xReact}}$ represents features extracted from *xReact*_*i*_.
  -
$o_{i}^{\text{A-oReact}}$ represents features extracted from *oReact*_*i*_.
  -
$o_{i}^{\text{C-xReact}}$ represents features extracted from *XGen*_*i*_.
  -
$o_{i}^{\text{C-oReact}}$ represents features extracted from *OGen*_*i*_.

For the knowledge fusion features extracted from *μ*_*i*_, we insert them as new nodes into the corresponding graph *G*_*i*_ at time *i*, forming a new graph $G_{i}^{*}$. The resulting graph not only represents the structure of the conversation well but also integrates relevant external commonsense knowledge. To obtain the final utterance features, we use a Graph Convolutional Network (GCN), which has demonstrated excellent performance in processing graph data [39, 40]. For the graph $G_{i}^{*}$ converted from text data, the GCN leverages the structural information within it to better understand the syntactic and semantic features of the sentences, thereby improving sentiment analysis performance. Additionally, by stacking multiple graph convolutional layers, it is possible to learn multi-level features of the text data layer by layer. The initial layers may capture lexical or local features, and as the number of layers increases, the model can capture deeper semantic features [41, 42]. This approach enhances the model’s understanding of complex semantic relationships and structures. Ultimately, each node in the graph is updated, and the updated nodes contain not only dialogue history information but also relevant commonsense knowledge. By stacking multiple Graph Convolutional Layers, the model can learn multi-level features of text data layer by layer. The initial layers may capture lexical or local features, and as the number of layers increases, the model can capture deeper semantic features. This approach enhances the model’s understanding of complex semantic relationships and structures.

### 3.4 Inference of emotions

The updated node $h_{t}^{*}$ from the GCN is used as the final discourse representation, where $h_{t}^{*}$ is the feature vector extracted from the discourse *μ*_*t*_ at time *t* through the GCN. To integrate the features extracted from previous layers and to enhance the model’s flexibility and adaptability, and to map these features to the target sentiment categories, we send them to a fully connected layer and apply softmax to obtain the final sentiment probability distribution, as shown below:

$$\begin{array}{c} E_{T}∼P\left(E_{T}|\left(\mu_{1},\mu_{2},\ldots ,\mu_{T}\right)\right)=\text{softmax}\left(W_{s}^{T}h_{T}^{*}+b\right). \end{array}$$ (4)

Here, $W_{s}^{T}$ is a learnable parameter matrix, and $h_{T}^{*}$ is the node embedding corresponding to the discourse *μ*_*T*_ after GCN updates. In the model training, we utilize the cross-entropy loss function for optimizing the trainable parameters. This loss function measures the difference between the probability distribution predicted by the model and the true label’s probability distribution, making the training objective and performance evaluation more intuitive. In this paper, it is defined as follows:

$$\begin{array}{c} L=-\frac{1}{N}\sum_{i=1}^{N}\sum_{c=1}^{C}\;y_{i,c}\text{log}\left(p_{i,c}\right), \end{array}$$ (5)

where *N* is the total number of samples, *C* is the total number of sentiment categories, *y*_*i*,*c*_ is the vector representation of the true sentiment category, *p*_*i*,*c*_ is the probability that the model predicts the *i*-th sample belongs to category *c*, and *L* is the average loss over the entire dataset. To minimize the difference between the predicted probability distribution of the model and the actual label’s probability distribution, the cross-entropy loss function is widely used in sentiment classification tasks to improve model accuracy. During this process, the model parameters are updated through the backpropagation algorithm to reduce the value of the loss function. Algorithm 1 describes the entire process of emotion inference using CEICG from a conversation dataset *U*.

**Algorithm 1** CEICG: emotion inference from a conversation dataset *U*

**Require:** a conversation dataset *U*

**Ensure:** the predicted emotional probability distribution *E*_*T*_

1: {*μ*_1_, *μ*_2_, …, *μ*_*T*_}←*U*

2: {*v*_1_, *v*_2_, …, *v*_*T*_} = *RoBERTa*(*μ*_1_, *μ*_2_, …, *μ*_*T*_)

3: **for** each *t* from 1 to *T* **do**

4:  *h*_*t*_ ← *LSTM*(*v*_*t*_, (*h*_*t*−1_, *c*_*t*−1_))

5: *H* ← {*h*_1_, *h*_2_, …, *h*_*T*_}

6: *γ* ← {{*h*_*i*_, *h*_*j*_}|*h*_*i*_, *h*_*j*_ ∈ *H*, *h*_*i*_ ≠ *h*_*j*_}

7: **for** each *t* from 1 to *T* **do**

8:  *G*_*t*_ ← (*H*_*t*_, *γ*_*t*_)

9:  *K*_*t*_ ← DGIEKGen(*G*_*t*_)

10:  $G_{t}^{*}\leftarrow insert\left(G_{t},K_{t}\right)$

11:  $h_{t}^{*}\leftarrow GCN\left(G_{t}^{*}\right)$

12: $E_{T}∼P\left(E_{T}|\left(\mu_{1},\mu_{2},\ldots ,\mu_{T}\right)\right)\leftarrow \text{softmax}\left(W_{s}^{T}h_{T}^{*}+b\right)$.

## 4 Analyses and results of experiments

### 4.1 Experimental datasets

The English dialogue datasets used in this experiment include:

- **MELD**: This dataset is a resource covering multi-party conversations [20], which includes text, audio, and video data derived from the TV show “Friends.” The dataset contains over 1,400 dialogues and more than 13,000 utterances. Each dialogue involves multiple participants, and each utterance is annotated with one of the following emotion labels: surprise, joy, disgust, anger, sadness, fear, and neutral. In this experiment, the predefined textual utterances and the pre-split training and test sets from MELD are used directly. The dataset can be accessed and downloaded via the following URL: https://affective-meld.github.io/
- **Topical Chat**: This is a topical chat dataset obtained from Amazon. It contains over 8,000 dialogues and 184,000 utterances. Each utterance is labeled with one of the following emotion labels: curious, happy, sad, surprised, neutral, and others. These labels represent the emotions perceived by the sender. The dataset can be accessed and downloaded via the following URL: https://github.com/alexa/Topical-Chat
- **DailyDialog**: This dataset covers a wide range of topics, including daily life, work, travel, health, finance, etc., aiming to simulate real daily conversation scenarios. In addition to the original dialogue text, the dataset also provides annotations of emotions and communicative intents for each sentence in the dialogue, supporting research in emotion analysis and intent recognition. The dataset comprises multi-turn dialogues that are carefully designed and written to ensure they are both natural and reflective of real-life conversation patterns. The dataset includes over 13,000 multi-turn dialogues, with emotional annotations based on Ekman’s six basic emotions. The dataset can be accessed and downloaded via the following URL: http://yanran.li/dailydialog.html

Some statistical information about these three datasets is shown in Table 2.

**Table 2:** Statistical results of the MELD, Topical Chat and DailyDialog datasets.

Dataset	Number of the Dialogues	Average Length of the Dialogues	Dialogues Type	Emotion Category
MELD	1424	12	Multi-person	7
Topical Chat	8136	24	Multi-person	6
DailyDialog	6942	9	Dual-person	6

### 4.2 Evaluation metrics

To evaluate the model’s performance, this experiment uses Precision, Recall, and the weighted F1-score as evaluation metrics. Precision measures the proportion of correctly predicted samples among all predicted results. Recall measures the proportion of correctly predicted samples among all actual samples. The F1-score is the harmonic mean of Precision and Recall. The formulas are as follows:

$$\begin{array}{c} precision=\frac{Num_{correct_{pre}}}{Num_{gold}}\times 100\%, \end{array}$$ (6)

$$\begin{array}{c} Recall=\frac{Num_{correct_{pre}}}{Num_{pre}}\times 100\%, \end{array}$$ (7)

$$\begin{array}{c} F1-score=2\times \frac{Precision\times Recall}{Precision+Recall}\times 100\%. \end{array}$$ (8)

Here, $Num_{correct_{pre}}$ represents the number of correctly predicted sentiments, *Num*_*gold*_ represents all samples predicted as that category (i.e., true positives and false positives)., and *Num*_*pre*_ refers to the sum of the number of correctly classified samples and the number of incorrectly classified samples.

### 4.3 Hyper parameters

In these experiments, the BERT layer uses the base version of RoBERTa with a hidden layer dimension of 768 and employs dropout for regularization. The LSTM hidden layer dimension is set to 128. The GCN consists of two layers, and the optimal number of fully connected layers is 2. The default number of training iterations is 1000, with the parameter optimization method using the Adam optimizer. The learning rate is set to 0.001, and the batch size is set to 64. Experimental environment: RTX 3090 with 24 GB of VRAM, CPU is i9–12900K, development platform is PyCharm, and the deep learning framework is PyTorch 1.13.2.

### 4.4 Baseline models

The proposed model is compared with six baseline models:

- **LSTM** [43]: This model uses a basic LSTM structure, which effectively captures long-distance dependencies by introducing three gates (forget gate, input gate, output gate) and a cell state. It ultimately obtains contextual representations of historical utterances from the conversation and uses an emotion classifier for emotion classification.
- **LSTM+ATT** [44]: This model combines LSTM with an attention mechanism. The attention mechanism helps the model identify words or sentences most crucial for emotion prediction, such as emotionally charged vocabulary, thereby improving prediction accuracy. It enhances the model’s performance by leveraging the ability of LSTM to process sequential data and remember long-term dependencies, along with the attention mechanism’s advantage of highlighting key information.
- **DialogueRNN** [45]: The DialogueRNN model is a recurrent neural network architecture specifically designed for dialogue systems, aimed at better capturing emotional dynamics and interaction relationships among participants in a conversation. Unlike traditional RNNs, DialogueRNN particularly considers dependencies between roles (such as speakers) and the dialogue context, to improve the accuracy and depth of tasks like emotion analysis and dialogue understanding.
- **DialogueGCN** [40] (Dialogue Graph Convolutional Networks): This model is a graph convolutional neural network specifically designed for dialogue systems, intended to capture complex structures and dynamic relationships within a conversation. By treating dialogue as a graph structure, where nodes represent utterances and edges denote various relationships between utterances (e.g., replies, references), DialogueGCN effectively understands and utilizes contextual information. Its advantage lies in its ability to integrate semantic information from the dialogue content and relational information from the dialogue structure, enhancing the dialogue system’s contextual understanding.
- **DialogInfer-Ensemble** [28]: This model focuses on emotion inference tasks in multi-turn dialogues by simulating the propagation of emotional states between speakers in the dialogue history. It introduces an address-aware module that can automatically learn whether participants in the next round of dialogue maintain historical emotional states or are influenced by others. Additionally, an ensemble strategy is proposed to extract multiple potential emotional responses, further enhancing model performance.
- **DialogueGLP** [29]: This method combines the temporal sequence processing abilities of RNNs with the deep language understanding capabilities of PLMs, enabling the model to deeply analyze dialogue history for more accurate predictions of upcoming emotional states, even in the absence of direct utterance information from the responder. It not only focuses on emotion expression in individual utterances but also considers the complex dynamics of emotion propagation and change in dialogues. Moreover, by inputting the entire dialogue history as contextual information into the PLM and using context learning to extract and generate knowledge, it provides a unique way for the model to understand and leverage the implicit deep semantics and emotional cues within the dialogue.

### 4.5 Analyses and results of experiments

#### RQ1: Performance comparison with existing models

**Fig 2:**
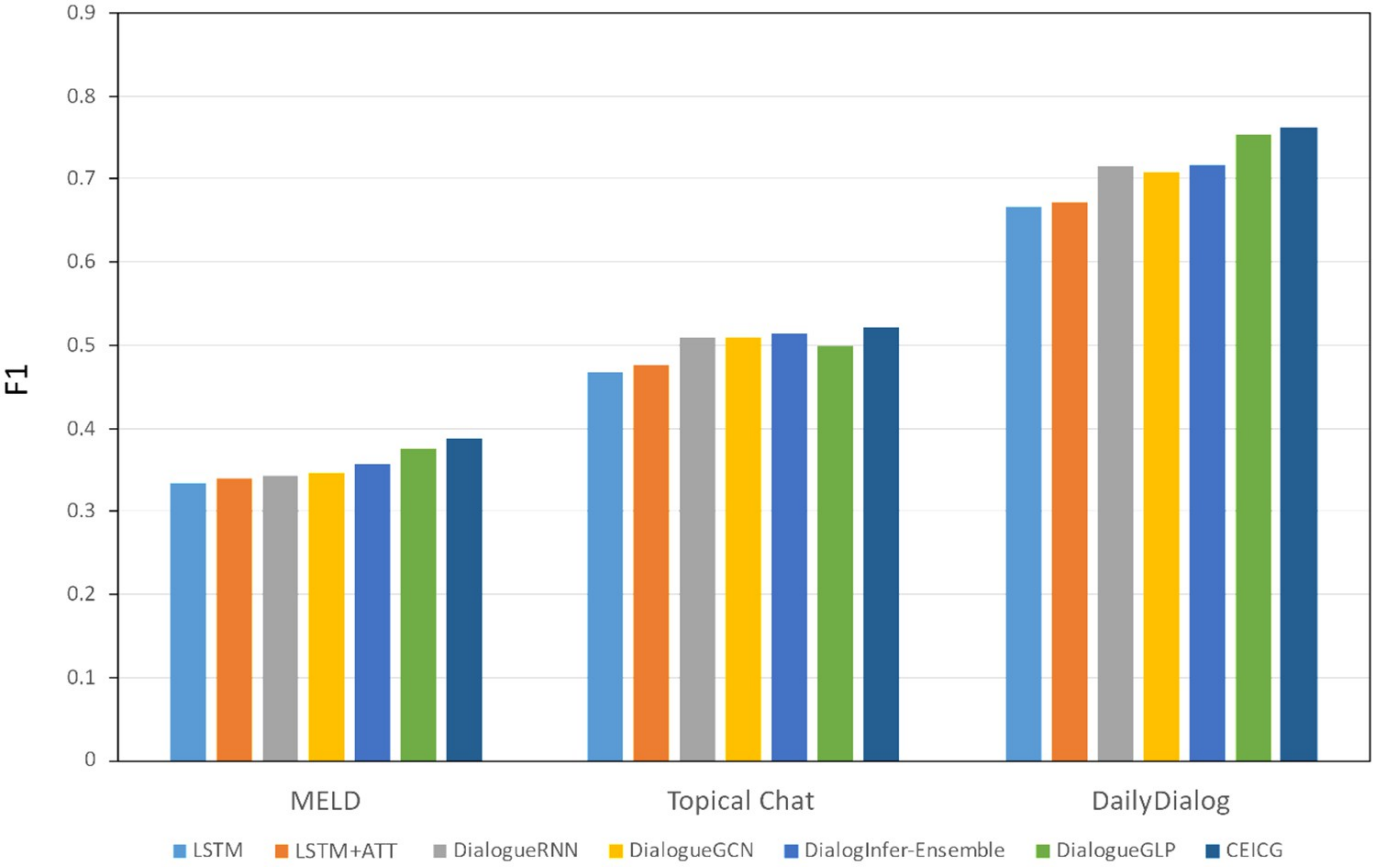
F1 score of emotion inference.

To verify the effectiveness of the proposed CEICG model in the emotion inference task, we conducted experimental comparisons between the CEICG model and the six baseline models mentioned above across three dialogue datasets. The experimental results are shown in Tables 3–5, and Fig 2.

**Table 3:** Experimental results of emotion inference on the DailyDialog dataset.

Model	Precision	Recall	F1-score
LSTM	0.6715	0.6624	0.6669
LSTM+ATT	0.6823	0.6618	0.6719
DialogueRNN	0.7167	0.7128	0.7147
DialogueGCN	0.7125	0.7034	0.7079
DialogInfer-Ensemble	0.7325	0.7027	0.7173
DialogueGLP	0.7913	0.7177	0.7527
CEICG	**0.8005**	**0.7289**	**0.7630**

**Table 4:** Experimental results of emotion inference on the Topical Chat dataset.

Model	Precision	Recall	F1-score
LSTM	0.4921	0.4456	0.4677
LSTM+ATT	0.5024	0.4522	0.4760
DialogueRNN	0.5424	0.4789	0.5087
DialogueGCN	0.5524	0.4729	0.5096
DialogInfer-Ensemble	0.5587	0.4754	0.5137
DialogueGLP	0.5438	0.4612	0.4991
CEICG	**0.5618**	**0.4867**	**0.5216**

**Table 5:** Experimental results of emotion inference on the MELD dataset.

Model	Precision	Recall	F1-score
LSTM	0.3432	0.3268	0.3348
LSTM+ATT	0.3463	0.3317	0.3388
DialogueRNN	0.3316	0.3543	0.3426
DialogueGCN	0.3416	0.3512	0.3463
DialogInfer-Ensemble	0.3713	0.3424	0.3563
DialogueGLP	0.3913	0.3623	0.3762
CEICG	**0.4036**	**0.3728**	**0.3876**

From Table 3, it can be seen that on the DailyDialog dataset, the precision of the CEICG model improved by 1.1% compared to the second-ranked DialogueGLP and by 19.2% compared to the lowest-ranked LSTM. The recall rate of CEICG improved by 1.6% compared to DialogueGLP and by 10.0% compared to LSTM. Its F1 score improved by 1.3% compared to DialogueGLP and by 14.4% compared to LSTM. The experiments indicate that, compared to the MELD and Topical Chat datasets, the DailyDialog dataset is characterized primarily by two-person conversations, with a greater number of dialogue turns (as shown in Table 2). Therefore, all models achieved better performance on this dataset. However, the performance of knowledge-based methods CEICG and DialogueGLP is superior to most models because the DailyDialog dataset consists of data generated from everyday conversations, which involves a broader range of external common knowledge compared to other datasets. Additionally, the dialogue length in the DailyDialog dataset is relatively shorter than that in long dialogue datasets, resulting in limited emotional features that can be extracted. Consequently, models based on historical emotional features tend to perform relatively weaker. In contrast, models that incorporate external common knowledge as auxiliary information allow us to capture more features for the emotion inference task, thereby enhancing the model’s performance.

As shown in Table 4, on the Topical Chat dataset, the precision of the CEICG model improved by 0.6% compared to the second-ranked DialogInfer-Ensemble and by 14.2% compared to the lowest-ranked LSTM. Its recall rate improved by 1.6% compared to the second-ranked DialogueRNN and by 9.2% compared to LSTM. The F1 score improved by 1.5% compared to DialogInfer-Ensemble and by 11.6% compared to LSTM. The experiments indicate that, compared to the DailyDialog dataset, the main characteristics of the Topical Chat dataset are fewer conversations and a multi-party dialogue format (as shown in Table 2). Consequently, all models performed at a lower level on this dataset. Among these models, LSTM still exhibited the worst performance, while DialogueGLP performed slightly worse than DialogInfer-Ensemble. This is because DialogInfer-Ensemble extracts features from the dialogue process based on both sequence and graph approaches, whereas the knowledge generated by DialogueGLP has weaker weights within the same topic. The experiments also demonstrate that, compared to the DailyDialog dataset, on the more challenging inference dataset Topical Chat, our model shows better advantages in terms of both precision and recall compared to the second-ranked DialogInfer-Ensemble.

From Table 5, it can be seen that in the MELD dataset, the accuracy of the CEICG model is improved by 3.1% compared to the second-ranked DialogueGLP and by 21.7% compared to the lowest-ranked Dialogue RNN. Its recall rate is improved by 2.8% compared to DialogueGLP and by 14.1% compared to the lowest-ranked LSTM. The F1 score of CEICG is improved by 3.0% compared to DialogueGLP and by 15.8% compared to the lowest-ranked LSTM. This experiment shows that although LSTM can effectively handle long-distance dependency issues, it often learns only superficial features from complex sentences. The LSTM+ATT model, with the added attention mechanism, allows the model to focus on sentences relevant to the emotion inference task, thereby reducing the impact of noise on the model. DialogueRNN enhances the ability to capture factors influencing emotion inference in multi-party dialogues by considering the characteristics of the speaker and the interlocutors, resulting in significant improvements compared to LSTM and LSTM+ATT, which rely solely on historical dialogue features. As for DialogueGCN and DialogInfer-Ensemble, both are based on sequence and graph models that transform the multi-party dialogue process into a graph node format, effectively simulating the dialogue evolution process, allowing the model to better understand the semantic and structural features of the conversation. This results in F1 score improvements of 0.00370 and 0.0137 over DialogueRNN, demonstrating the feasibility of converting the dialogue process into a graph structure. DialogueGLP, by incorporating external knowledge through a pre-trained model, shows some improvement over DialogueGCN and DialogInfer-Ensemble. Experimental results prove that our model CEICG, through the definition of graph models with different relational edges and the extraction of external knowledge via knowledge queries and fusion, better accomplishes the emotion inference task.

The comparison of the F1 scores for emotion inference across the three datasets is shown in Fig 2. It can be observed that on the DailyDialog dataset, the F1 scores of all models are relatively high, with the proposed CEICG model showing a certain advantage over the second-ranked DialogueGLP. On the more challenging Topical Chat dataset, the proposed CEICG model exhibits a greater advantage compared to the second-ranked DialogInfer. Furthermore, on the most difficult MELD dataset, the CEICG model has an absolute advantage over the second-ranked DialogueGLP. Additionally, compared to all other models, the proposed model demonstrates robust performance, adapting well to more complex real-world data environments.

#### RQ2:Ablation experiments

**Fig 3:**
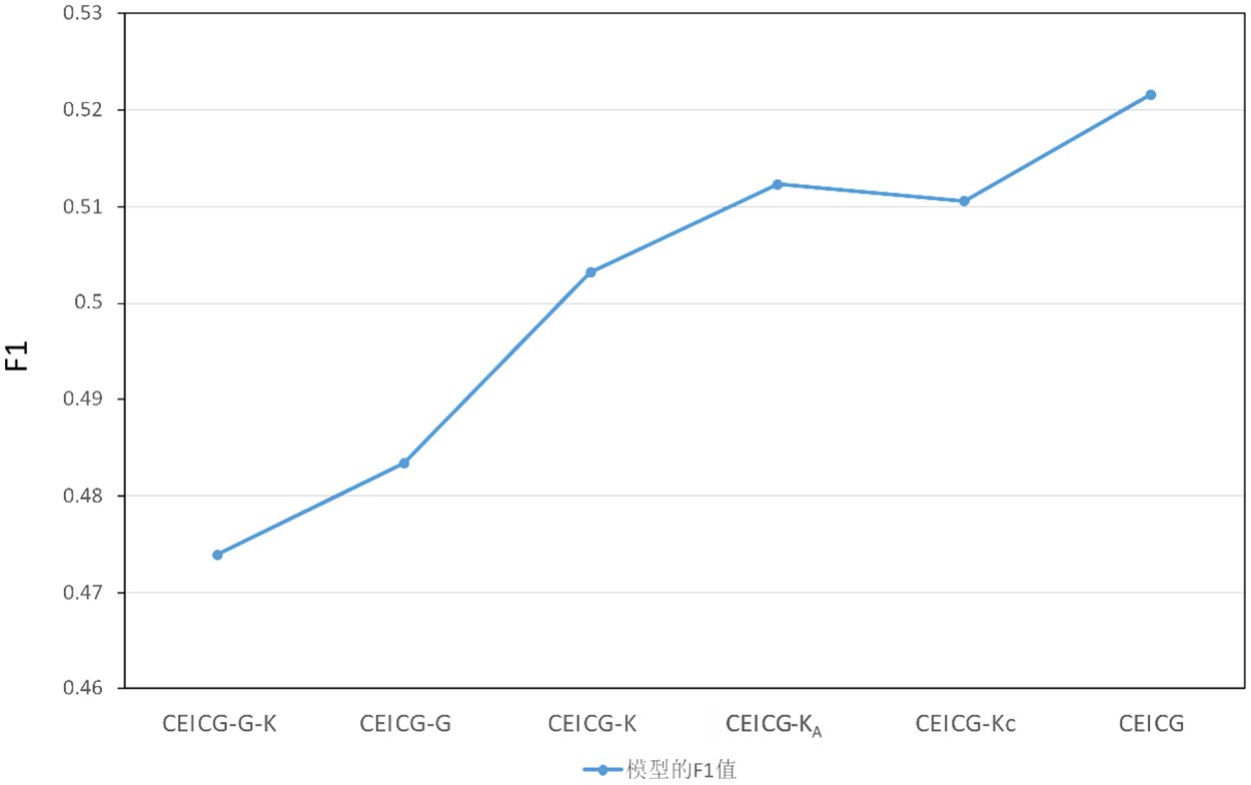
The evaluation score of the ablation methods.

To understand the impact of the various components of the model on the final performance, we systematically removed or modified specific parts of the CEICG model and observed how these changes affected the model’s performance, thereby validating the effectiveness of each module in the CEICG model. This experiment was based on the Topical Chat dataset, and the results are shown in Table 6 and Fig 3. In Table 6, “-” indicates the removal of the corresponding structure. CEICG-G-K refers to the CEICG model with the graph model and external knowledge component removed; CEICG-G refers to the model with only the graph component removed, integrating external knowledge into the text feature vector; CEICG-K refers to the model with the external knowledge component removed; CEICG-KA refers to the model with the external knowledge module’s knowledge query structure removed; and CEICG-KC refers to the model with the external knowledge module’s knowledge generation structure removed.

**Table 6:** The results of ablation experiment.

Model	Precision	Recall	F1-score
CEICG-G-K	0.4987	0.4517	0.4739
CEICG-G	0.5038	0.4645	0.4834
CEICG-K	0.5348	0.4752	0.5032
CEICG-KA	0.5401	**0.4873**	0.5123
CEICG-Kc	0.5478	0.4782	0.5106
CEICG	**0.5618**	0.4867	**0.5216**

The results indicate that whether removing the graph model or the external knowledge module, the model experiences a corresponding decline in performance. The performance decreased by 3.82% when only the graph model was removed, demonstrating that utilizing a graph structure to represent the dialogue process aids the model in better understanding the structural and semantic features of the conversation, leading to the greatest improvement in model performance. When only the external knowledge component was removed, the performance decreased by 1.84%. Although this impact is not as significant as that of the graph model, it still suggests that incorporating external knowledge as auxiliary information helps enhance the model’s understanding capabilities. Additionally, we analyzed the roles of knowledge querying and knowledge generation within the knowledge fusion component. From the experimental results, both parts contribute to the model’s performance enhancement. These findings reveal that the various components of our model play a crucial role in the task of emotion reasoning, highlighting the importance of each component within the model.

## 5 Conclusions

This paper presents a new CEICG model for the emotion reasoning task, which primarily leverages external knowledge and graph models for emotion inference. The model first utilizes RoBERTa to embed dialogue sentences, obtaining rich contextual representations. LSTM is then employed for extracting emotional features, which are used to create nodes in the graph model. The edges of the graph model are constructed based on the sequential and semantic characteristics of the nodes. External knowledge is obtained through knowledge querying and knowledge generation, which is then integrated into the graph model. Finally, Graph Convolutional Networks (GCN) are used to update the nodes in the graph model, followed by classification using an emotion classifier. A comparison with several existing baseline methods demonstrates that the CEICG model achieves competitive results in the emotion reasoning task, with F1 scores surpassing those of baseline methods across three datasets. Particularly, for the daily dataset, which involves a wide range of common sense knowledge but consists of shorter sentences, our model demonstrates superior performance.

## Supporting information

S1 File Data set MELD. **Description:** Public dataset that was used in this study, including all measured variables. **Format:** pkl file. **Availability:** The complete dataset can be downloaded from the following link: URL: https://affective-meld.github.io/.
(ZIP)

S2 File Data set topical chat. **Description:** Public dataset that was used in this study, including all measured variables. This dataset can be directly downloaded from the internet. **Format:** json file. **Availability:** The complete dataset can be downloaded from the following link: https://github.com/alexa/Topical-Chat.
(ZIP)

S3 File Data set dailyDialog. **Description:** Public dataset that was used in this study, including all measured variables. **Format:** txt file. **Availability:** The complete dataset can be downloaded from the following link: http://yanran.li/dailydialog.html.
(ZIP)
